# Abdominal organ position variation in children during image-guided radiotherapy

**DOI:** 10.1186/s13014-018-1108-9

**Published:** 2018-09-12

**Authors:** Sophie C. Huijskens, Irma W. E. M. van Dijk, Jorrit Visser, Brian V. Balgobind, D. te Lindert, Coen R. N. Rasch, Tanja Alderliesten, Arjan Bel

**Affiliations:** Amsterdam UMC, University of Amsterdam, Department of Radiation Oncology, Cancer Center Amsterdam, Meibergdreef 9, Amsterdam, The Netherlands

**Keywords:** Interfractional organ position variation, Abdominal organ motion, Pediatric RT, IGRT

## Abstract

**Background:**

Interfractional organ position variation might differ for abdominal organs and this could have consequences for defining safety margins. Therefore, the purpose of this study is to quantify interfractional position variations of abdominal organs in children in order to investigate possible correlations between abdominal organs and determine whether position variation is location-dependent.

**Methods:**

For 20 children (2.2–17.8 years), we retrospectively analyzed 113 CBCTs acquired during the treatment course, which were registered to the reference CT to assess interfractional position variation of the liver, spleen, kidneys, and both diaphragm domes. Organ position variation was assessed in three orthogonal directions and relative to the bony anatomy. Diaphragm dome position variation was assessed in the cranial-caudal (CC) direction only*.* We investigated possible correlations between position variations of the organs (Spearman’s correlation test, ρ), and tested if organ position variations in the CC direction are related to the diaphragm dome position variations (linear regression analysis, R^2^) (both tests: significance level *p* < 0.05). Differences of variations of systematic (∑) and random errors (σ) between organs were tested (Bonferroni significance level *p* < 0.004).

**Results:**

In all directions, correlations between liver and spleen position variations, and between right and left kidney position variations were weak (ρ ≤ 0.43). In the CC direction, the position variations of the right and left diaphragm domes were significantly, and stronger, correlated with position variations of the liver (R^2^ = 0.55) and spleen (R^2^ = 0.63), respectively, compared to the right (R^2^ = 0.00) and left kidney (R^2^ = 0.25). Differences in ∑ and σ between all organs were small and insignificant.

**Conclusions:**

No (strong) correlations between interfractional position variations of abdominal organs in children were observed. From present results, we concluded that diaphragm dome position variations could be more representative for superiorly located abdominal (liver, spleen) organ position variations than for inferiorly located (kidneys) organ position variations. Differences of systematic and random errors between abdominal organs were small, suggesting that for margin definitions, there was insufficient evidence of a dependence of organ position variation on anatomical location.

**Electronic supplementary material:**

The online version of this article (10.1186/s13014-018-1108-9) contains supplementary material, which is available to authorized users.

## Background

Continuous developments in pediatric cancer treatment using multimodality strategies, including surgery, chemotherapy, and radiotherapy have led to increasing numbers of childhood cancer survivors [1]. Inevitably, the occurrence of treatment associated adverse events has also increased. Treatments including radiotherapy significantly contribute to the risk of developing adverse events.

Children are treated with abdominal and thoracic radiotherapy for a wide range of primary cancer diagnoses, including Wilms’ tumor, neuroblastoma, and Ewing sarcoma. Moreover, treatment of the craniospinal axis and lung metastasis involve irradiation of the abdominal and thoracic region. The anatomical locations of these tumors and adjacent organs at risk (OARs) vary; target volumes can be in very close proximity to the lungs, diaphragm, liver, spleen, and kidneys. As a result, healthy tissues and OARs are unavoidably exposed to radiation when irradiating the tumor [2, 3]. Although adequate tumor dose coverage is the primary goal in radiotherapy, sparing the vital and long-term functions of adjacent organs is also of great concern. Especially in children, who have a relative long life expectancy when surviving cancer, organs are still growing and have low tolerance to radiation [4, 5]. To ensure adequate tumor dose coverage while minimizing radiation dose to surrounding healthy tissues, knowledge about the extent of target and organ motion, particularly present in the abdominal and thoracic area, is needed. Thus, quantifying the motion of vital and sensitive organs such as the liver, spleen, and kidneys is essential.

These abdominal organs move with every breathing cycle (intrafraction motion) and from day-to-day (interfraction motion). Intra- and interfractional motion of the tumor and OARs are incorporated by expanding the clinical target volume and OARs volumes to the planning target volume (PTV) and planning risk volumes (PRVs), respectively [6]. In adults, many studies have quantified motion of various organs, enabling to define accurate margins for PTVs and PRVs. Despite the increasing number of publications on pediatric organ motion [7–14], data is still limited and no consensus has been reached in pediatric radiotherapy to define PTV or PRV margins for abdominal tumors or OARs. Therefore, PTV margins for children are currently pragmatically based on available adult data and PRV margins are often not used in pediatric radiotherapy. Due to different anatomical locations (e.g., right vs. left side of the abdomen, (retro)peritoneum, adjacent to the vertebrae), or abdominal processes (e.g., intestinal peristaltic or air pockets), abdominal organ motion might be location-dependent, as was discussed before in Van Dijk et al. [14]. This could lead to differences in PTV and PRV margins depending on the anatomical location.

The most commonly used PTV margin recipe is from van Herk et al. (2.5 ∑ + 0.7 σ), where the systematic (∑) and random (σ) component are based on quadratically adding the systematic/random errors that occur during treatment ($\sqrt{\Sigma_{\operatorname{int} er}^2+\kern0.5em {\Sigma}_{\operatorname{int} ra}^2}$ and $\sqrt{\sigma_{\operatorname{int} er}^2+\kern0.5em {\sigma}_{\operatorname{int} ra}^2}$) [15]. Previous studies mainly reported on *intra*fractional organ motion, focusing on respiratory-induced abdominal organ motion through various phases of the breathing cycle as measured on a single four-dimensional computed tomography (4DCT) [9, 11, 16] or 4D magnetic resonance imaging (4DMRI) [12, 17]. Although organ motion seems to be more prone to respiratory motion than to day-to-day position variations, Guerreiro et al. showed that in a homogenous group of 15 children, interfractional abdominal organ motion was larger than intrafraction motion (Σ_int*er*_ and *σ*_int*er*_ > Σ_int*ra*_ and *σ*_int*ra*_) [16]. In addition, Huijskens et al. showed that for respiratory-induced diaphragm motion in children the systematic error was found to be smaller than the random error (Σ_int*ra*_ < *σ*_int*ra*_) [8]. This seems to indicate that the systematic component of the PTV and PRV margins is predominated by the day-to-day systematic (i.e., interfractional) variations (Σ_int*er*_). Moreover, van Herk’s margin recipe shows that the systematic component weighs more than the random component [15]. Therefore, quantification and a comprehensive understanding of interfractional abdominal organ motion is essential for high-accuracy image-guided radiotherapy.

Most studies on abdominal organ motion have focused only on the quantification of the *inter*fractional component [7, 10, 16, 18], without investigating location-dependency, or possible correlations between organ position variations. Whenever possible, resection of a tumor takes place before radiation treatment and usually surgical clips are placed to localize the remaining tumor bed. If not, an anatomical structure close to the target could function as a surrogate for localization and position variation. However, such a strategy will only be successful when there is a clear understanding of the correlations between the tumor and the anatomical surrogate. In addition, radiation treatment might also lead in the future towards adaptive strategies in children. However, often, certain organs are not directly visible on daily cone beam CTs (CBCTs), due to artefacts, smaller field of view or, especially in children, low dose imaging protocols. Moreover, markers are rather not placed in children and online evaluation of the positions of organs is thus mostly unfeasible in clinical practice. Here as well, another close anatomical structure might be considered as a surrogate. For instance, when the diaphragm, being very well visible on CBCT images, is used as a surrogate for the assessment of abdominal organ position. Some adult studies have shown reliable correlations of the diaphragm with abdominal organs [19–22], while other studies show weak correlations [20, 23–25]. This is mostly depending on the tumor site and therefore, outcomes cannot be generalized for adults. For children, correlations between the diaphragm and abdominal organs has not been extensively studied. It is therefore crucial to have a clear understanding of the correlation between the tumor or organ and a particular surrogate.

Therefore, the aim of this study was to increase the insight on interfractional position variation of abdominal organs in children. We investigated possible correlations between abdominal organs and determined whether position variation is location-dependent. Additionally, we investigated whether diaphragm position variation could be a surrogate for abdominal organ position variation, by analyzing the right and left diaphragm domes separately.

## Methods

### Patient population

**Fig. 1:**
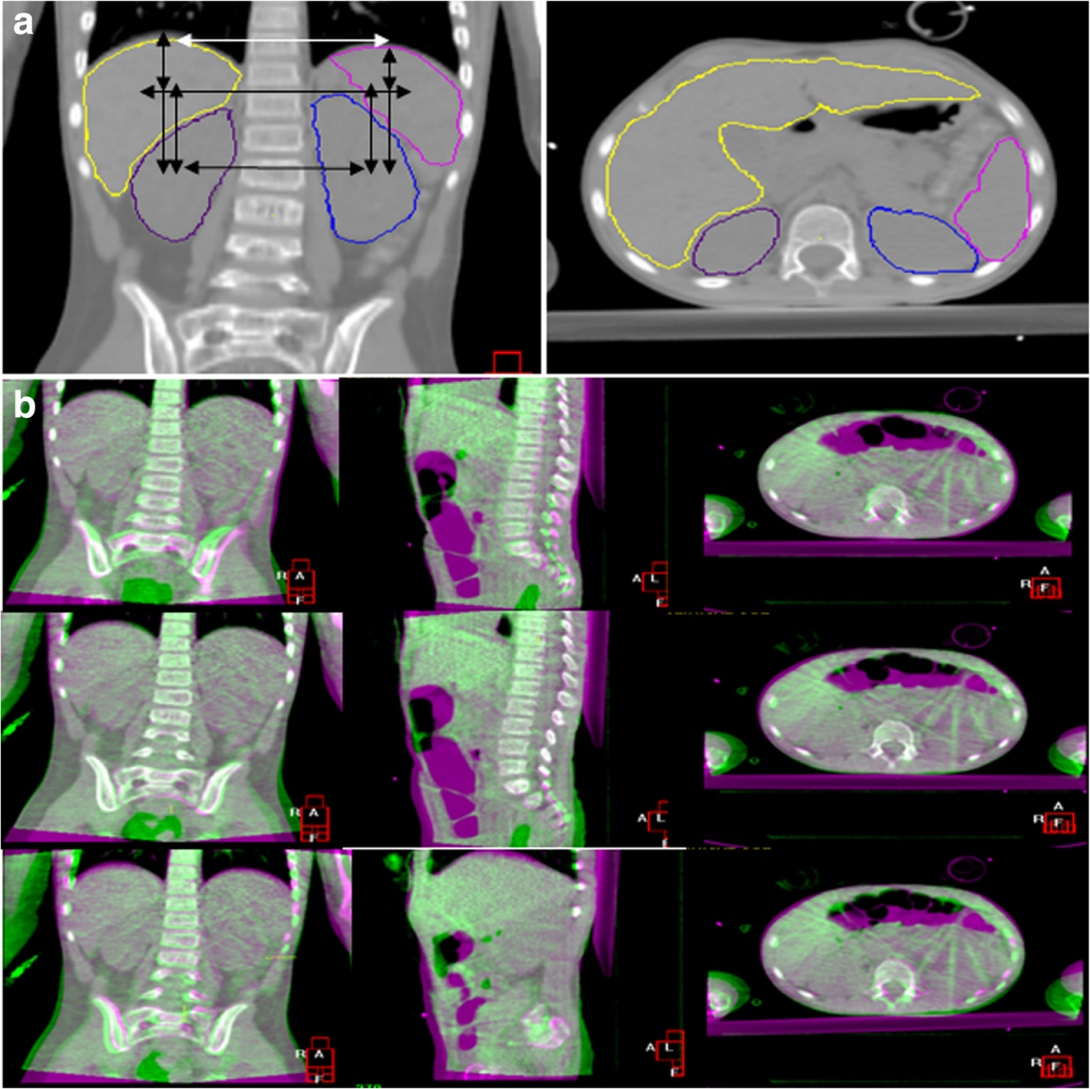
**a** Delineated organs (right kidney: purple, left kidney: blue, liver: yellow, spleen: pink) on the reference CT. Diaphragm domes are not delineated. Arrows indicate mutual correlations investigated. **b** Example of the two-step rigid registration (from top to bottom): unaligned overlap of reference CT and CBCT, bones aligned, right kidney aligned (note: bones shifted). (Color figure online only)

For this retrospective study, we included 20 patients younger than 18 years, treated for various tumors at our radiation oncology department between December 2010 and September 2017 (Table 1). Patients were included if a pre-treatment CT scan and multiple CBCT scans of the abdomen or thorax were available, in which the liver, spleen, kidneys, and right and left diaphragm domes were visible (Fig. 1).

**Table 1:** Patient characteristics

No.	Sex	Tumor	Age at diagnosis (years)	Height (cm)	Weight (kg)	No. of CBCTs	RT location
1	F	Sarcoma	11.5	155	38	5	Thorax
2	M	Medulloblastoma	6.6	110	18	5	Spinal cord^a^
3	F	Hodgkin lymphoma	16.5	166	49	5	abdomen
4	M	Medulloblastoma	14.1	175	36	5	Spinal cord
5	M	Medulloblastoma	8.3	128	25	5	Spinal cord
6	F	Medulloblastoma	6.7	117	20	2	Spinal cord
7	M	Ewing sarcoma	16.8	184	62	8	Thorax
8	M	Medulloblastoma	6.7	129	24	5	Spinal cord
9	M	Spinal metastesis	2.6	90	12	8	Thorax
10	F	Medulloblastoma	7	118	22	6	Spinal cord
11	M	Anaplastic glioma	7.9	132	31	5	Spinal cord
12^b^	M	Medulloblastoma	5.1	109	17	8	Spinal cord
13	F	Neuroblastoma	5.3	115	24	6	Abdomen
14	M	Sarcoma	10.9	142	37	5	Thorax
15	M	DSRCT	9.9	137	26	5	Abdomen
16	M	Neuroblastoma	4.7	118	22	6	Abdomen
17^b^	M	Medulloblastoma	4.9	105	18	6	Spinal cord
18	M	Ewing sarcoma	17.9	182	81	7	Thorax
19	F	Osteosarcoma	15.1	159	53	5	Thorax
20	M	Neuroblastoma	2.2	90	15	6	Abdomen

*Abbreviations*: *M* male, *F* female, *DSRCT* desmoplastic small round cell tumor

^a^ Spinal cord was part of craniospinal irradiation

^b^ Patients 12 and 17 were treated under general anesthesia.; this did not influence interfractional organ position variations

### Imaging data

For each patient, a pre-treatment CT scan (120 kV, 2.5- or 5 mm slice thickness) was acquired for planning purposes (LightSpeed RT16; General Electric Company, Waukesha, WI, USA). This scan was considered as the reference CT (refCT) scan and included original organ delineations, as used for clinical practice (Fig. 1). For all patients, CBCT images (1 mm slice thickness, 1 mm in-plane resolution) were routinely acquired using the CBCT scanner mounted on the Elekta Synergy linac (Elekta AB, Stockholm, Sweden) as part of the position verification protocol. This yields CBCT imaging at the first three treatment fractions, followed by daily or weekly CBCT acquisitions, depending on the treatment protocol. To be consistent, we included for all patients the first three CBCTs and thereafter weekly acquired CBCTs. All CBCTs were acquired with 120 kV, 10 mA, and 10 or 40 ms exposure time per projection. The scanning time of the CBCT scan varied between 35–60s, and the degree of circumferential rotation was 200 or 360 degrees. In this study, we retrospectively analyzed the imaging data, including a total of 20 refCTs and 113 CBCTs.

### Imaging registration

Elekta X-ray Volume Imaging software (XVI 3.0; Elekta AB, Stockholm, Sweden) was used for a two-step rigid registration for each organ separately (example shown in Fig. 1). First, a region of interest (ROI) was defined in the refCT, including the 12th thoracic through the 4th lumbar vertebra (from the lowest part of the kidneys up to the diaphragm domes). The CBCTs were then registered to the refCT using the automatic chamfer match algorithm [26]. Second, this bony anatomy-based match was followed by registration of each organ separately (i.e., liver, spleen, right kidney, left kidney) with a grey value algorithm [26], based on shaped ROIs defined by the delineated organs including (at least 2/3rd of) the whole organ volume. This enabled the assessment of organ position variation smaller than the slice thickness of the acquired refCT. Automatic registration outcomes (translations and rotations) were visually checked (by SCH/DTL) and manually corrected if necessary. Results were corrected for rotations as follows. First, we assessed the center of mass (COM) coordinates for each organ. Then, we equated these coordinates to the refCT to determine the exact magnitude and direction of the interfractional position variation. By calculating the difference of the magnitude and sign of the COM coordinates of each organ on CBCTs and refCT, registrations resulted in interfractional position variation relative to bony anatomy, expressed as composite vectors in the left-right (LR), cranio-caudal (CC) and anterior-posterior (AP) directions*.* The + and – signs respectively indicate right/caudal/posterior and left/cranial/anterior directions**.** For the diaphragm, the bony anatomy-based automatic chamfer match was followed by manual registrations of the right- and left-sided diaphragm dome separately in the CC direction only (by SCH/DTL).

### Statistical analysis

For each patient, organ specific mean and standard deviation (SD) of the interfractional position variation relative to the bony anatomy were determined in the three orthogonal directions, and in the CC direction only for the right and left diaphragm domes. Furthermore, over all patients, we estimated per organ the group mean (i.e., mean of the individual means), the group systematic error (∑; the SD of the individual means of all patients), and the group random error (σ; the root mean square of the individual SDs of all patients).

To evaluate whether organ position variation is location-dependent, we compared contralateral and superiorly and inferiorly located abdominal organs separately (indicated in Fig. 1). Since not all data fitted a normal distribution (tested with the Shapiro-Wilk’s test), differences between contralateral organs’ systematic and random errors were separately tested (i.e., right diaphragm dome vs. left diaphragm dome, liver vs. spleen, right kidney vs. left kidney) with the Levene’s test (for ∑) and Mann-Whitney U-test (for σ). Also, differences in ∑ and σ between superiorly and inferiorly located abdominal organs were tested (i.e., liver vs. right kidney, spleen vs. left kidney). Since differences were tested in 14 combinations (i.e., LR, CC, AP for four organs, and CC only for both diaphragm domes), we adjusted *p* values according to the Bonferroni correction. Differences were considered to be significant if test outcomes showed a *p* value< 0.004 (i.e., 0.05/14).

We used the Spearman’s correlation test (significance level *p < 0.05*) to investigate the possible correlations in position variations between contralateral organs.

Additionally, to test if right- and left-sided organ position variations in the CC direction are related to the position variations of the superiorly located right- and left-sided diaphragm dome respectively, we used linear regression analysis (significance level *p < 0.05*). Both tests were also performed for each individual patient.

All statistical analyses were done using R version 3.2.1. (R Foundation for Statistical Computing, USA).

## Results

**Fig. 2:**
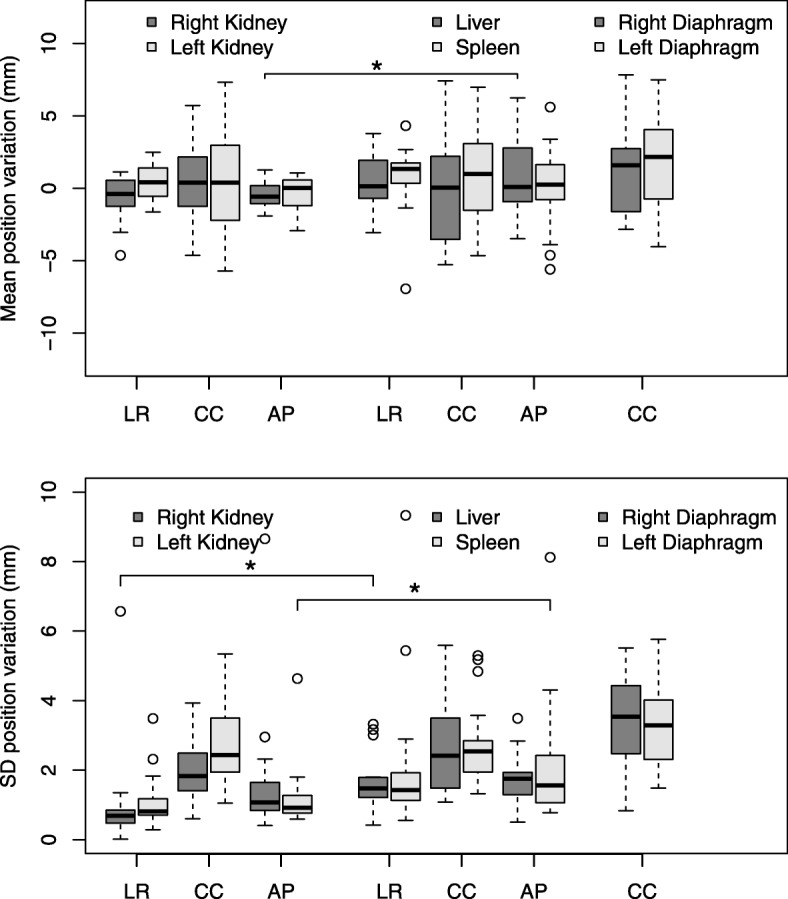
Boxplots showing the distributions of the individual means (upper panel) and SDs (lower panel) of the interfractional position variations found for right- (light grey) and left-sided (dark grey) organs for all 20 patients. Horizontal bars, boxes, and whiskers represent medians, 50th percentiles (inter quartile range (IQR)), and the highest (lowest) value within 1.5xIQR, respectively. Circles denote outliers. *Significant differences (Bonferroni corrected *p < 0.004*). Abbreviations: LR = left–right; CC = cranial–caudal; AP = anterior–posterior

Mean organ position variation was smaller than 1.0 mm (range: − 6.9 to 7.4 mm) for the abdominal organs in all orthogonal directions and smaller than 1.8 mm (range: − 4.0 to 7.8 mm) for the diaphragm domes in the CC direction. For all organs and across all fractions, ranges of position variations were largest in the CC direction (varying from 10.6 to 13.0 mm) and smallest in the LR direction (varying from 4.1 to 11.1 mm) (Fig. 2). Overall, kidney position variations were smaller than position variations of the liver and spleen (Fig. 2). Table 2 presents the values of the group mean, systematic and random error per organ in each direction, mainly showing average systematic error in decreasing order of CC (3.2 mm, SD = 0.3 mm), AP (1.9 mm, SD = 0.9 mm), and LR (1.7 mm, SD = 0.5 mm) direction, and average random error also in decreasing order of CC (3.0 mm, SD =0.5 mm), AP (2.1 mm, SD = 0.6 mm), and LR (1.9 mm, SD = 0.7 mm) direction. Differences of the systematic error between right- and left-sided organs were insignificant (*p ≥* 0.004), as were the differences of the random error between right- and left-sided organs (*p ≥* 0.004) (Additional file 1: Table S1). For superiorly and inferiorly located organs, significant but small differences were found between the liver and the right kidney in the AP direction (*p =* 0.002 for ∑), and in the LR direction (*p =* 0.000 for σ). Also, the random error of the spleen and the left kidney was significantly different in the AP direction (*p =* 0.001) (Additional file 1: Table S1).

**Table 2:** The group systematic (Σ) and group random errors (σ) in mm in the orthogonal directions for the right kidney, left kidney, liver, and spleen and in CC direction for the diaphragm

(mm)	Right Kidney			Left Kidney			Liver			Spleen			Right Diaphragm	Left Diaphragm
	LR	CC	AP	LR	CC	AP	LR	CC	AP	LR	CC	AP	CC	CC
Group mean	−0.6	0.7	−0.4	0.4	0.4	−0.4	0.4	−0.1	1.0	0.8	0.8	0.0	1.2	1.8
Σ	1.4	2.8	0.9	1.1	3.3	1.3	2.1	3.4	2.7	2.2	3.5	2.7	3.0	3.4
σ	1.6	2.2	2.4	1.3	2.9	1.4	1.8	2.8	1.9	2.8	3.0	2.7	3.6	3.4

*Abbreviations*: *LR* left–right, *CC* cranial–caudal, *AP* anterior–posterior

**Fig. 3:**
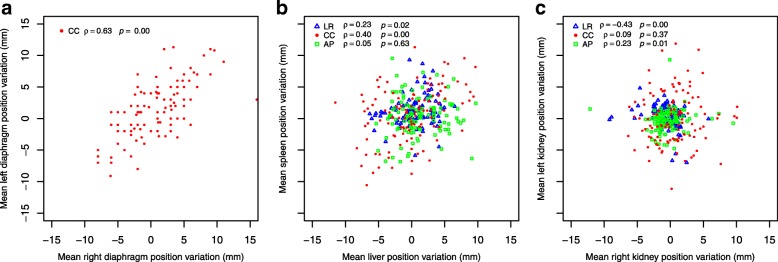
For all CBCT scans, scatterplots describing relations (Spearman’s ρ and *p*-value) between right and left diaphragm position variations in the CC direction only (**a**) and right- (x-axis) and left-sided (y-axis) organ interfractional position variations separately (**b**; liver and spleen, **c**; right and left kidney), in the three orthogonal directions. (Color figure online only). Abbreviations: LR = left–right; CC = cranial–caudal; AP = anterior–posterior

A moderate and statistically significantly correlation between the position variations of the right and left diaphragm domes was found (ρ = 0.63, *p =* 0.00) (Fig. 3a). The position variations of the liver and spleen in the LR and CC direction were weakly, but statistically significantly correlated (ρ = 0.23, *p* = 0.02 and ρ = 0.40, *p* = 0.00, respectively) (Fig. 3b). Position variations of the right and left kidney were weakly, but statistically significant correlated in the LR and AP directions (ρ = − 0.43, *p* = 0.00 and ρ = 0.23, *p* = 0.01, respectively) (Fig. 3c). Correlations within each individual patient were similar to the overall group outcomes.

**Fig. 4:**
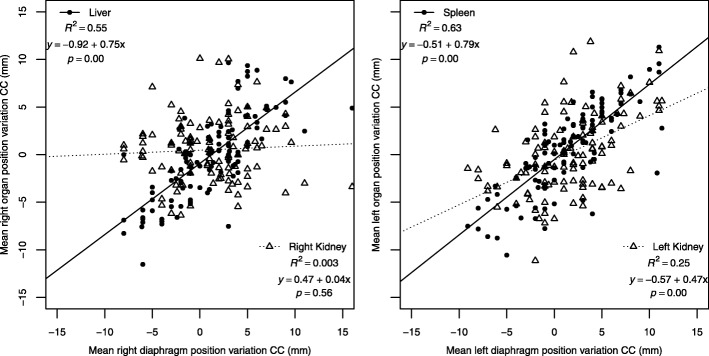
Scatterplots with regression lines of the linear regression analyses describing relationships for each CBCT between right- and left-sided interfractional organ position variation (y-axis) and diaphragmatic position variation in the CC direction (x-axis). Abbreviations: CC = cranial–caudal

Linear regression analysis showed that right and left diaphragm dome position variations in the CC direction were significantly correlated with position variations of the liver (R^2^ = 0.55, *p* = 0.00) and spleen (R^2^ = 0.63, *p* = 0.00), respectively. In the CC direction, no (strong) correlation was found between right and left diaphragm dome position variations and the position variations of the right (R^2^ = 0.003, *p* = 0.60) and left kidney (R^2^ = 0.25, *p* = 0.00), respectively (Fig. 4).

## Discussion

In this study, we quantified interfractional position variation of multiple abdominal organs in 20 children during radiotherapy and evaluated if organ position variation is mutually related and location-dependent. We found weak correlations between the position variations of contralateral organs. In the CC direction, right and left diaphragm dome position variations correlated moderately with the position variations of the liver and spleen, respectively. However, correlations between the position variations of the diaphragm domes and those of both kidneys were negligible. Furthermore, the largest magnitude of organ position variations was observed in the CC direction, followed by the AP and LR directions. We found that differences between group systematic and random errors of abdominal organs were small and insignificant. This comprehensive analysis of organ position variations at different anatomical locations increases the insight in possible consequences for margin definitions, which has not been reported on for children so far.

Nazmy et al. studied interfractional position variation of the liver and kidneys in 9 children (mean age: 4.1 years, SD = 1.6 years) using reference CT and CBCT scans [10]. They also found that, in the CC direction, the liver showed more motion than the kidneys. However, their range of observed position variations of the left kidney was smaller than that of the right kidney. In contrary, when we analyzed patients in our cohort of similar age (*n* = 6; range 2.2–5.3 years) we found slightly larger position variations of the left kidney compared to the right kidney. Although this comparison involves small sample sizes, a possible explanation might be the different methodology in choosing the point of interest. Nazmy et al. used the upper pole of the kidneys whereby kidney deformations might have been interpreted as translations, resulting in an overestimation of motion. We used the COM as point of interest because it is less sensitive to organ deformations. Although data on organ deformation would provide useful additional information on organ motion characteristics, analyzing organ deformation was outside the scope of the current study.

Our results are comparable to findings of Guerreiro et al. who used a similar methodology as we did [16]. They quantified interfractional position variations of the spleen, liver, and the healthy kidney in patients (*n* = 15, mean age: 4 years) with Wilms’ tumors. Their ranges of interfractional position variation, and the systematic and random errors were generally somewhat smaller than our results, which could be explained by the fact that their cohort consisted of younger patients (age range 1–8 years). However, when we analyzed patients in our cohort of similar age (*n* = 10; range 2.2–7.8 years), the systematic and random errors for all organs and directions in our cohort remained somewhat larger (for Σ; mean difference 1.0 mm, SD = 0.6 mm, for σ; mean difference 0.7 mm, SD = 0.7 mm).

Using a 3DCT as a reference point to estimate interfractional position variation is arguable. The 3DCT represents ‘snapshot’ of repeatedly changing organ positions during the respiratory cycle [27]. A CBCT captures in 35–60 s several complete respiratory cycles and averages the motion over the observed breathing phases into one blurred 3D image. To investigate the possible effect of respiratory motion differences on the 3DCT and the CBCTs, we recalculated our measurements using the first CBCT scan as the reference scan instead of the 3DCT. Differences between the respective calculations based on the refCT and the first CBCT were negligible (< 1 mm). Also, although projection images could enable the quantification of intrafractional motion as well [28], the low dose CBCT protocols that we used for most children [29] unavoidably result in poorer quality of projection images. Therefore, we were not able to distinguish organs on the two-dimensional projection images of these CBCT scans in order to investigate intrafractional motion of the liver, spleen, and kidneys.

The outliers shown in Fig. 2 represent the SD values of the right kidney and spleen position variations of three patients. For one patient, in which the field of view of the CBCT scan was smaller than its refCT, the whole right kidney was visible on the refCT but remained only half visible on the CBCT scan, and registration was performed using an adjusted sub-volume of the kidney. Additionally, in this patient the distance of the COM of the right kidney to the treatment planning isocentre on the refCT was relatively large (> 10 mm), resulting in a large deviation in organ position variation. For two other patients, the two-step rigid registration for the spleen yielded large rotations (> 15 degrees), resulting in large ranges of position variations. However, a sensitivity analysis, excluding these three cases, did not change our results.

The liver and spleen are contralateral organs that substantially differ in tissue composition and function. However, regarding their position variations, differences were small and position variations of both organs were moderately correlated with the position variations of the diaphragm domes. In contrary, the position variations of both kidneys were smaller and showed weak correlations with the diaphragm dome position variations. This might be due to their more inferior and retroperitoneal location. Further, visual inspection showed that the kidneys seem more prone to deformations than the liver and spleen, probably due to their different tissue composition. Therefore, although in the CC direction only, diaphragm position variations seem to particularly be more representative for position variations of OARs in the upper abdomen than for OAR position variations in the lower abdomen.

The weak to moderate (ρ < 0.4), however significant, correlations of position variations between right- and left-sided abdominal organs suggest that organs move only somewhat in similar directions. Therefore, for future online strategies, close located anatomical structures are not recommended as suitable surrogates. However, the overall magnitude of motion is small, and differences of systematic and random errors of the various abdominal organs are small and insignificant, hence negligible. Therefore, regarding margin definitions, there was insufficient evidence of a dependence of organ position variation on anatomical location. Additionally, although differences between abdominal organ position variations were small, overall position variation was largest in the CC direction and smallest in the LR direction. This suggests that margins should be applied anisotropically rather than isotropically. Note, however, that the diaphragm was measured in the CC direction only.

Knowledge about patient’s day-to-day anatomical variation is furthermore valuable when (automating) selecting similar patients from a database of patients’ CT scans for, e.g., automating treatment planning or dose reconstruction [30–34], because this provides a lower bound on the achievable precision of selection.

Besides, as recommended by the Paediatric Radiation Oncology Society (PROS), consensus needs to be reached regarding appropriate margin definitions in children [35]. With increasing data, knowledge on organ motion during radiotherapy in children is expanding. However, due to generally small patient numbers and different methodologies in separates studies, definitive statements regarding margin definitions cannot be made yet. Therefore, close collaborations between research groups, and pooling of data might contribute to achieving consensus on margin definitions. A summarized all-encompassing overview of all published data so far, including inter- and intrafractional organ motion, could provide a basis for this. Especially, with more proton and carbon therapy facilities in development, aiming for high-precision radiotherapy and the need for the assessment of the anatomical variations in children, induced by organ motion, becomes even more important.

## Conclusions

No (strong) correlations between interfractional position variations of abdominal organs in children were observed. Differences of systematic and random errors between abdominal organs were small, suggesting that for margin definitions, there was insufficient evidence of a dependence of organ position variation on anatomical location. From present results, we concluded that diaphragm dome position variations could be more representative for superiorly located abdominal (liver, spleen) organ position variations than for inferiorly located (kidneys) organ position variations.

## Additional file

Additional file 1: **Table S1.** *p*-values for differences of group systematic errors (Σ) and group random errors (σ); tested for differences between right- and left-sided organs and superiorly vs. inferiorly located organs in the orthogonal directions. (DOCX 16 kb)

## Abbreviations

3DCT: 3-Dimensional computed tomography
4DCT: 4-Dimensional computed tomography
4DMRI: 4-Dimensional Magnetic Resonance Imaging
AP: Anterior-Posterior
CBCT: Cone Beam Computed Tomography
CC: Cranial-Caudal
COM: Center of Mass
CT: Computed Tomography
LR: Left-Right
OAR: Organ at Risk
PROS: Paediatric Radiation Oncology Society
PRV: Planning Risk Volume
PTV: Planning Target Volume
SD: Standard Deviation
