# Supplementary material for: Abdominal organ position variation in children during image-guided radiotherapy
Source: Radiat Oncol. 2018 Sep 12;13:173. doi: 10.1186/s13014-018-1108-9 (PMC6136223; doi:10.1186/s13014-018-1108-9)
Supplement: Supplementary file 1 — Table S1. p-values for differences of group systematic errors (Σ) and group random errors (σ); tested for differences between right- and left-sided organs and superiorly vs. inferiorly located organs in the orthogonal directions. (DOCX 16 kb) [file 13014_2018_1108_MOESM1_ESM.docx]

| **Table S1.** P-values for differences of group systematic errors (Σ) and group random errors (σ); tested for differences between right- and left-sided organs and superiorly vs. inferiorly located organs in the orthogonal directions. | | | | | | | | | | | | | | | | | |
| --- | --- | --- | --- | --- | --- | --- | --- | --- | --- | --- | --- | --- | --- | --- | --- | --- | --- |
|  | Right Kidney vs. Left Kidney | | |  | Liver vs. Spleen | | |  | Right Kidney vs. Liver | | |  | Left Kidney vs. Spleen | | |  | Right Diaphragm vs. Left Diaphragm |
| LR | CC | AP |  | LR | CC | AP |  | LR | CC | AP |  | LR | CC | AP |  | CC |  |
| Σ ^a^ | 0.66 | 0.46 | 0.17 |  | 0.58 | 0.97 | 0.67 |  | 0.14 | 0.34 | **0.002*** |  | 0.42 | 0.82 | 0.08 |  | 0.66 |
| σ ^b^ | 0.05 | 0.05 | 0.21 |  | 0.89 | 0.38 | 0.914 |  | **0.00*** | 0.27 | 0.07 |  | 0.02 | 0.77 | **0.001*** |  | 0.62 |

^a^ Differences tested with the Levene’s test

^b^ Differences tested with the Mann-Whitney U-test

* adjusted *p* values according to the Bonferroni correction (Significance level : *p* value<0.004 (i.e., 0.05/14))
